# Racial inequality in health care of adults hospitalized with COVID-19

**DOI:** 10.1590/0102-311XEN215222

**Published:** 2023-11-13

**Authors:** Fernanda Sandes Cardoso, Danilo Cosme Klein Gomes, Alexandre Sousa da Silva

**Affiliations:** 1 Universidade Federal do Estado do Rio de Janeiro, Rio de Janeiro, Brasil.; 2 Universidade do Estado do Rio de Janeiro, Rio de Janeiro, Brasil.

**Keywords:** COVID-19, Hospital Mortality, Race Factors, Health Services Accessibility, COVID-19, Mortalidade Hospitalar, Fatores Raciais, Acesso aos Serviços de Saúde, COVID-19, Mortalidad Hospitalaria, Factores Raciales, Accesibilidad a los Servicios de Salud

## Abstract

The objective was to analyze the association of race/skin color in health care, in adults hospitalized with severe acute respiratory syndrome (SARS)/COVID-19, between March 2020 and September 2022, with Brazil as the unit of analysis. This is a cross-sectional study that used the Influenza Epidemiological Surveillance Information System (SIVEP-Gripe) database and had a population composed of adults (≥ 18 years) and the final classification was SARS by COVID-19 or unspecified SARS. The direct effect of skin color on in-hospital mortality was estimated through logistic regression adjusted for age, gender, schooling level, health care system and period, stratified by vaccination status. This same model was also used to assess the effect of skin color on the variables related to access to health care services: intensive care unit (ICU), tomography, chest X-ray and ventilatory support. The results show that black, brown and indigenous people died more, regardless the schooling level and number of comorbidities, with 23%, 32% and 80% higher chances of death, respectively, when submitted to ventilatory support. Racial differences were observed in the use of health care services and in outcomes of death from COVID-19 or unspecified SARS, in which ethnic minorities had higher in-hospital mortality and lower use of hospital resources. These results suggest that black and indigenous populations have severe disadvantages compared to the white population, facing barriers to access health care services in the context of the COVID-19 pandemic.

## Resumo

O objetivo deste artigo foi analisar a associação entre raça/cor e assistência à saúde, em adultos hospitalizados pela síndrome respiratória aguda grave (SRAG)/COVID-19 no Brasil, entre março de 2020 e setembro de 2022. Trata-se de estudo transversal, que utilizou o banco de dados do Sistema de Informação de Vigilância Epidemiológica da Gripe (SIVEP-Gripe) e contou com uma população composta por adultos (≥ 18 anos). A classificação final foi SRAG por COVID-19 ou SRAG não especificada. O efeito direto do aspecto cor na mortalidade intra-hospitalar foi estimado por meio de regressão logística ajustada por idade, sexo, escolaridade, sistema de saúde e período, estratificado por situação vacinal. Esse mesmo modelo foi utilizado também para avaliar o efeito do quesito cor nas variáveis de acesso aos serviços de saúde: unidade de terapia intensiva (UTI), tomografia, radiografia de tórax e suporte ventilatório. Os resultados evidenciam que pretos, pardos e indígenas morreram mais, independentemente do grau de escolaridade e da quantidade de comorbidades, com maiores chances de óbito em 23%, 32% e 80%, respectivamente, ao serem submetidos ao suporte ventilatório. Foram observadas diferenças raciais no uso de serviços de saúde e nos desfechos de morte por COVID-19 ou SRAG não especificada, em que minorias étnicas tiveram maiores taxas de mortalidade intra-hospitalar e os recursos hospitalares foram utilizados com menos frequência. Tais resultados sugerem que as populações negra e indígena têm severas desvantagens em relação à branca, enfrentando barreiras de acesso aos serviços de saúde no contexto da pandemia de COVID-19.

## Resumen

Se pretende analizar la asociación entre raza/color en la atención de la salud de adultos hospitalizados por síndrome respiratorio agudo severo (SARS)/COVID-19 en el período entre marzo de 2020 y septiembre de 2022, tomando Brasil como unidad de análisis. Se trata de un estudio transversal, en que se utilizó la base de datos del Sistema de Información de Vigilancia Epidemiológica de la Gripe (SIVEP-Gripe) y tuvo una muestra compuesta por adultos (≥ 18 años) y la clasificación final fue SARS por COVID-19 o SARS no especificado. El efecto directo de la pregunta color sobre la mortalidad intrahospitalaria se estimó mediante la regresión logística ajustada según edad, sexo, nivel de estudios, sistema de salud y período, estratificada por estado de vacunación. Este modelo también se utilizó para evaluar el efecto de la pregunta color en las variables de acceso a los servicios de salud: unidades de cuidado intensivo (UCI), tomografía, radiografía de tórax y soporte ventilatorio. Los resultados muestran que negros, pardos e indígenas murieron más, independientemente del nivel de estudios y el número de comorbilidades, y cuando fueron sometidos a soporte ventilatorio, tuvieron mayores probabilidades de muerte con el 23%, 32% y 80%, respectivamente. Se observaron diferencias raciales en el uso de los servicios de salud y los resultados de muerte por COVID-19 o SARS no especificado, en los que las minorías étnicas tuvieron una mayor mortalidad intrahospitalaria y los recursos hospitalarios se utilizaron con menos frecuencia. Estos resultados evidencian que las poblaciones negra e indígena tienen graves desventajas en comparación con la población blanca, afrontando dificultades de acceso a los servicios de salud en el contexto de la pandemia del COVID-19.

## Introduction

On March 11, 2020, the World Health Organization (WHO) declared a pandemic state due to the large number of infections caused by the new coronavirus ^1^. One of the clinical manifestations of COVID-19 is the development of severe acute respiratory syndrome (SARS) ^1^ ^,^ ^2^.

Globally, around 600 million cases of the disease have been reported, with more than 6 million deaths reported to date ^3^. The high number of deaths during the pandemic period has a direct or indirect association with COVID-19, mainly concentrated in Southeast Asia, Europe and the Americas. Low- and middle-income countries represent 53% of the 14.9 million excess deaths in the period of 2020-2021, with higher rates of death among males and the elderly ^4^ ^,^ ^5^.

Black people, Asian people, and ethnic minorities have an increased risk of death from COVID-19 ^6^. A systematic review with 54 studies published in 2020 ^7^, on racial and ethnic disparities related to the disease, observed that African-American/black populations have a 1.5 to 3.5 times higher risk of COVID-19 infection compared to white populations, for hospitalization the risk was 1.5 to 3 times higher, while mortality was 3.2 times higher in African-American/black populations.

In the United States in 2020, a study observed that mostly black counties are, respectively, 3 and 6 times more likely to have infection and death caused by the disease, compared to counties with mostly white people ^8^. Another study conducted in England in 2020 ^9^, using the UK Biobank as source, pointed out that among black participants the risk of death from COVID-19 was about 7 times higher (odds ratio - OR = 7.25; 95% confidence interval - 95%CI: 4.65-11.33), compared to Asian participants (OR = 1.98; 95%CI: 1.02-3.84) and with the white population as reference.

In Brazil, the largest and most populous country in Latin America, estimates indicate 35 million people infected and 686,000 killed by COVID-19 as of October 12, 2022 ^10^. Although it has the second largest black population in the world (about 54%), in the country it remains marginalized, with precarious access to health care, diagnosis and treatment resources, resulting in more significant impacts in the pandemic context ^11^ ^,^ ^12^. Recent studies have found that race and ethnicity are identified as risk factors for hospitalization due to COVID-19, reinforcing the existing disparities ^13^ ^,^ ^14^.

A retrospective analysis of adult patients hospitalized with COVID-19 in Brazil, with data obtained from the Influenza Epidemiological Surveillance Information System (SIVEP-Gripe) database, showed lower use of hospital resources and more serious conditions for black patients compared to white patients. Black patients had higher in-hospital mortality, after adjusting for gender, age, schooling level, region of residence and comorbidities ^15^.

Thus, this study aims to analyze the association between race/skin color and access and health care in adults hospitalized due to SARS/COVID-19, in health care institutions in Brazil in 2020, 2021 and 2022.

## Material and method

SIVEP-Gripe is the main database for monitoring cases of and deaths from SARS in Brazil, including additional information on sociodemographic variables, clinical symptoms, comorbidities, laboratory tests, vaccination history and hospitalization outcomes (death or discharge) ^16^. The database is available free of charge at the website: https://opendatasus.saude.gov.br/dataset/srag-2021-a-2023. SARS was defined as a flu-like syndrome with dyspnea/respiratory distress or persistent chest pressure or oxygen saturation of less than 95% in ambient air or bluish color of lips or face ^2^.

### Study design and population

This is a cross-sectional study with nationwide data on hospitalization due to SARS/COVID-19 in Brazil. The SIVEP-Gripe database was created by the Brazilian Ministry of Health in 2009 for the monitoring of influenza A (H1N1). In March 2020, the system included cases of SARS due to COVID-19 throughout the Brazilian territory. The notification of SARS in Brazil is mandatory within 24 hours after the identification of the case in the public and private health care network ^16^.

Hospitals were classified as public or private according to their financial source. This information was obtained from the Brazilian National Register of Health Establishments (CNES). This is the official Brazilian register of all health units in the country. The CNES database is available free of charge at the website: http://tabnet.fiocruz.br/dash/menu_dash.htm. In Brazil, health care is provided by the government to all, with a large proportion of people using public health care services ^17^.

The study population was composed of adults (≥ 18 years) hospitalized due to SARS between March 2020 and September 2022, with final classification of cases as SARS by COVID-19 or unspecified SARS, that is, cases in which no other etiological cause was confirmed. These cases were clinically and epidemiologically attributed to COVID-19. We excluded non-hospitalized SARS, SARS from other confirmed causes and in the variable “evolution” we excluded “deaths from other causes” and “ignored”, resulting in a final number of 2,459,844.

### Study variables

The main outcome was in-hospital mortality (death). Among the sociodemographic variables, the following were considered: race/skin color, self-reported as white, black, brown, yellow or indigenous; gender (female and male); age (continuous); and schooling level (illiterate, elementary school 1st cycle, elementary school 2nd cycle, high school, and higher education).

The comorbidities considered were: obesity, diabetes and cardiovascular disease. The variable received values from 0 to 3 according to the occurrence of one of the comorbidities. The information on comorbidities was self-reported or diagnosed directly by a health care professional. The professional could report it in two ways: by a specific dichotomous variable (yes or no) or by an open field variable.

For the open field variable, individuals who were described with terms in Portuguese: “*obesidade*”, “*obsidade*”, “*obeso*” ou “*obesa*” were considered obese in our study, although the dichotomous variable (obese: yes or no) was filled with none or absent.

The same scheme was used for diabetes and cardiovascular disease. The terms “*diab*” and “*dm*” were used to improve diabetes information, while for cardiovascular disease the terms were “*has*”, “*h.a.s*”, “*ic*”, “*ard*”, “*hiperte*”, “*iam*”, “*infart*”, “*fa*”, “*fibril*”, “*ts*”, “*hipote*”, “*fallot*”, “*bavt*”, “*mitral*”, “*prolaps*”, “*revasc*”, “*chaga*”, “*bradi*”, “*artero*”, or “*marcapasso*”. This variable does not include only patients with systemic arterial hypertension, but also patients with several other cardiovascular comorbidities such as: acute myocardial infarction, cardiac arrhythmias, valvular diseases, congenital or structural heart diseases, Chagas disease, among others.

To assess the use of health instruments, we considered dichotomous variables, namely: intensive care unit (ICU) hospitalization (yes or no), use of ventilatory support (yes - invasive and non-invasive - or no), chest X-ray (yes - normal, interstitial infiltrate, consolidation, mixed, other - or not performed), tomography (yes - typical COVID-19, indeterminate COVID-19, atypical COVID-19, negative for pneumonia, other - or not performed).

Information on the type of health care service (public or private), considered as a dichotomous variable, was also considered. To consider the time of the pandemic when hospitalization occurred (March 2020 to September 2022), we considered the information of the Epidemiological Week of the first symptoms and created a period variable with nine categories. Each category corresponded to 16 sequential Epidemiological Weeks, the first category started on March 1, 2020 and the last category was composed of three weeks.

### Statistical analysis

In-hospital mortality (frequencies and rates) was estimated according to sociodemographic variables, comorbidities and health care system. The effect of the race/skin color variable on in-hospital mortality was estimated through logistic regression adjusted for age (continuous), gender, schooling level, year (2020, 2021 and 2022) and health care system (public or private). This same model was also used to assess the effect of race/skin color on the use of health care resources, with these models having the following outcomes: ICU, tomography, chest X-ray and ventilatory support.

For a more in-depth analysis of the effect of race/skin color on in-hospital mortality, the logistic model was considered having the death variable (yes or no) as outcome; this model was stratified for those who stayed in the ICU, underwent tomography, chest X-ray, hospitalized patients who received ventilatory support, year, health care system and period. All analyses were performed in the R program, version 2021.09.2 (http://www.r-project.org), and the statistical significance considered was 0.05.

## Results

A total of 2,459,844 hospitalized individuals were analyzed. The mean age was 61 years (standard deviation - SD = 17.8), and 54.4% were male. Regarding schooling level, of the 866,455 patients who had this information recorded, 7.2% were illiterate and 13.8% had higher education. For 2,021,991 (82.2%) of the individuals who had information on race/skin color, 52.5% declared themselves white, 40.5% brown, 5.6% black, 1.21% yellow, 0.21% indigenous.

Regarding information on comorbidity, 959,339 (39%) people had this field filled in and, of these, 18% did not have any comorbidities (obesity, diabetes and cardiovascular diseases), 48.5% had one of the comorbidities, 27.2% two, and 5.5% three comorbidities. For 87.5% of the individuals there was information on the type of health care service (public or private) and of these 81.5% were hospitalized in the public health care system.

Regarding the use of health resources, 35.7% stayed in the ICU, information on whether or not they had stayed in the ICU was available for 89.5% of the total number of hospitalized patients. Of the total of 86.5% of patients with information on the use of ventilatory support, 77.5% received ventilatory support (invasive or non-invasive). For 53% of the total there was information on whether or not they underwent chest X-ray examination and, of these, 623,735 (48%) underwent the examination. Information on whether or not tomography was performed was available for 1,305,260 of the total hospitalized patients and of these 71% underwent tomography.

Table 1 shows in-hospital mortality stratified by race/skin color. Of illiterate individuals, 42.45% of the white people had an outcome of death, while for black people it was 47.43%, for browns it was 48.03% and 46.96% indigenous people. Individuals with higher education were 22.8%, 26.72% and 31.3% respectively for white, black and indigenous people. Among white women 29.39% died, among black women 34.18% and among indigenous women 28.02% died. Regarding white men, 31.17% died, among black men 34.93%, and indigenous men 31.3%. In patients without comorbidities, 31.52% of white and 32.71% of black people died.

**Table 1:** In-hospital mortality stratified by race/skin color. Brazil, March 2020 to September 2022.

Variables	White (%)	Black (%)	Yellow (%)	Brown (%)	Indigenous (%)
Year					
2020	29.38	34.10	31.18	33.07	33.71
2021	31.41	35.55	28.24	32.17	31.75
2022	27.88	31.21	28.34	28.85	24.61
Schooling level					
Illiterate	42.45	47.43	39.16	48.03	46.96
Elementary school 1st cycle	38.19	40.49	35.34	41.02	34.95
Elementary school 2nd cycle	32.99	35.59	30.49	35.01	36.06
High school	25.65	28.59	24.77	27.04	24.53
Higher education	22.80	26.72	21.75	25.52	31.30
Sex					
Female	29.39	34.18	27.67	31.09	28.02
Male	31.17	34.93	30.82	33.16	35.66
Comorbidities					
0	31.52	32.71	30.92	32.27	33.57
1	32.84	34.36	30.95	33.72	37.78
2	38.23	40.70	36.83	39.86	44.61
3	43.45	45.75	40.46	44.99	48.28
Health care service					
Private	23.00	24.57	22.52	22.38	15.87
Public	31.46	35.37	31.18	33.16	32.91

Regarding the health care service, in the public system, 31.46% of white, 35.37% black, 31.18% yellow, 33.16% brown and 32.91% indigenous people died. Of the patients who had access to the ICU and died, 53.82% were white, 57.19% black, 52.19% yellow, 56.65% brown and 59.53% indigenous people. For ventilatory support, the highest mortality rates are among black (40.28%) and indigenous people (40.71%), the same is repeated for tomography (black 32.83%, indigenous 32.54%) and chest X-ray (black 34%, indigenous 34.05%) (Table 2).

**Table 2:** In-hospital mortality of the variables related to the use of health care services, stratified by race/skin color. Brazil, March 2020 to September 2022.

Variables	White (%)	Black (%)	Yellow (%)	Brown (%)	Indigenous (%)
ICU	53.82	57.19	52.19	56.65	59.53
Ventilatory support	35.55	40.28	34.93	38.10	40.71
Tomography	28.84	32.83	28.49	30.75	32.54
Chest X-ray	32.36	34.00	29.38	32.30	34.05

ICU: intensive care unit.

In the logistic regression model, adjusted for age, schooling level, gender, number of comorbidities, year and health care system, the risk of death by COVID-19 in black people is (OR = 1.21; 95%CI: 1.18-1.25), yellow people (OR = 0.98; 95%CI: 0.91-1.05), brown people (OR = 1.26; 95%CI: 1.25-1.28) and indigenous (OR = 1.67; 95%CI: 1.42-1.97), in all cases the comparison group is white. Table 3 presents the model with the same adjustments, but using the following as outcome variables: ICU stay, tomography, chest X-ray and use of ventilatory support.

**Table 3:** Results of the logistic models * with the following as outcome variables **: intensive care unit (ICU), tomography and chest X-ray and ventilatory support. Brazil, March 2020 to September 2022.

Variables	Black		Yellow		Brown		Indigenous	
	OR	95%CI	OR	95%CI	OR	95%CI	OR	95%CI
ICU	1.11	1.08-1.15	1.12	1.04-1.20	1.01	1.00-1.03	0.83	0.69-0.98
Tomography	0.72	0.70-0.74	0.83	0.77-0.89	0.67	0.66-0.69	0.86	0.72-1.01
Chest X-ray	1.10	1.06-1.13	0.87	0.80-0.93	0.92	0.90-0.93	0.93	0.79-1.10
Ventilatory support	0.89	0.86-0.92	0.83	0.77-0.90	0.80	0.79-0.82	0.86	0.72-1.03

95%CI: 95% confidence interval; OR: odds ratio.

* For each of the outcomes, a logistic model was considered as adjustment variables: race/skin color, year, age, schooling level, sex, number of comorbidities and health care service;

** In the outcomes, the following were considered: whether stayed in the ICU, whether underwent tomography and chest X-ray, and whether received ventilatory support.

Black and yellow people have a significantly higher chance of staying in the ICU than white people, and for brown and indigenous people the statistical significance was borderline. White patients had more access to tomography compared to all others. Black patients were 10% more likely to undergo chest X-ray than white people. White patients were more likely to receive ventilatory support, being not significant for indigenous people.

Table 4 presents the results of the logistic regression model, adjusted for age, schooling level, gender, number of comorbidities, year and health care system, with response variable death by COVID-19. The model was stratified by year (2020, 2021, 2022), health care system (public and private) and the variables of use of health care services: stay in the ICU, tomography, chest X-ray and use of ventilatory support. In 2020, indigenous people were almost two times more likely to die than white people (OR = 1.99; 95%CI: 1.59-2.48). Indigenous individuals who use the public health care system had OR = 1.68 (95%CI: 1.42-1.97). In the private health care system, black people are 33% more likely to die than white people.

**Table 4:** Result of logistic models * with death outcome stratified ** for: intensive care unite (ICU) stay, tomography and chest X-ray and use of ventilatory support, year (2020, 2021, 2022), and health care service (public and private). Brazil, March 2020 to September 2022.

Variables	Black		Yellow		Brown		Indigenous	
	OR	95%CI	OR	95%CI	OR	95%CI	OR	95%CI
ICU	1.13	1.08-1.19	0.89	0.79-0.99	1.24	1.21-1.27	1.47	1.08-2.02
Tomography	1.15	1.10-1.21	0.91	0.81-1.02	1.28	1.25-1.32	1.88	1.43-2.46
Ventilatory support	1.23	1.19-1.27	0.98	0.90-1.07	1.32	1.29-1.34	1.80	1.49-2.17
Chest X-ray	1.22	1.16-1.28	0.85	0.74-0.97	1.25	1.22-1.29	1.61	1.23-2.10
2020	1.30	1.24-1.36	1.11	0.99-1.24	1.39	1.36-1.43	1.99	1.59-2.48
2021	1.16	1.11-1.21	0.87	0.78-0.97	1.19	1.17-1.22	1.60	1.21-2.10
2022	1.32	1.19-1.46	1.20	0.94-1.51	1.28	1.22-1.35	1.29	0.69-2.33
Public service	1.20	1.17-1.24	0.98	0.90-1.06	1.27	1.25-1.29	1.68	1.42-1.97
Private service	1.33	1.21-1.46	0.95	0.80-1.14	1.23	1.17-1.29	0.83	0.12-3.95

95%CI: 95% confidence interval; OR: odds ratio.

* For each of the outcomes, a logistic model was considered as adjustment variables: race/skin color, year, age, schooling level, sex, number of comorbidities and health care service;

** In the stratifications, we considered patients who: stayed in the ICU, underwent tomography and chest X-ray and received ventilatory support, hospitalized in 2020, 2021 and 2022, those who used the public service and those who used the private service.

**Figure 1:**
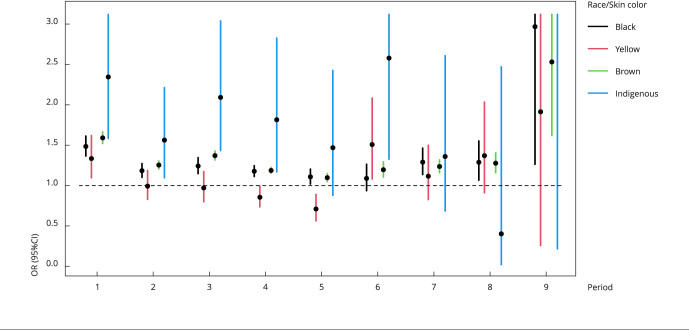
Risk of death from COVID-19 stratified by period. Brazil, March 2020 to September 2022.

Among hospitalized patients who stayed in the ICU, black people had a 13% higher risk of dying, for indigenous people the risk was 47%. For those who underwent tomography examination, indigenous people were 88% more likely to die compared to white people, for black and brown people the chances were respectively 15% and 28%. Among those who received ventilatory support, indigenous people had an 80% higher chance of dying, with risks of 23% and 32% for black and brown people, respectively. Among those who underwent chest X-rays, black people were 22% more likely to die than white people, 61% than indigenous people, and 25% than brown people. Figure 1 shows the risk of death from COVID-19 (OR and 95%CI) stratified by period, with a linear cutoff for values greater than 3 in the 95%CI.

## Discussion

We assessed the relation between race/skin color and in-hospital mortality of 2,459,844 patients hospitalized in Brazil with COVID-19. Of our study population, 81.5% were hospitalized in public health care system institutions, 86.5% were submitted to ventilatory support and 89.5% stayed in the ICU, and racial differences were observed in the in-hospital mortality rate.

In this context, black, brown and indigenous people were the patients who died the most, regardless of the schooling level and the number of comorbidities. These findings reinforce the maintenance of historical inequities and inequalities in the country, and especially, of structural racism, which dictates the way in which political, economic, legal and family relations are constituted, resulting in the constant marginalization of these populations in society ^18^.

The black population remains with the lowest wages in the labor market; the highest illiteracy rates; housing with lacking or precarious basic infrastructure services; higher levels of poverty; and even greater difficulties in accessing health care services ^19^ ^,^ ^20^.

Among the indigenous population, the situation is similar, and studies show that they are at a disadvantage in several sociodemographic and health indicators ^21^ ^,^ ^22^ ^,^ ^23^. This group also faces political, social and economic obstacles related to land tenure, exploitation of natural resources and implementation of development projects, directly linked to its process of illness and death ^23^ ^,^ ^24^ ^,^ ^25^ ^,^ ^26^.

In the COVID-19 context, there was a higher number of deaths from the virus among the black population. According to data from the Center for Healthcare Operations and Intelligence/Pontifical Catholic University of Rio de Janeiro (NOIS/PUC-Rio), black and brown people represent 55% of deaths from COVID-19, compared to 38% of deaths among white people ^27^ ^,^ ^28^.

A study conducted by the Coordination of the Indigenous Organizations of the Brazilian Amazon (COIAB) and the Amazon Environmental Research Institute (IPAM) also showed that the mortality rate from COVID-19 among indigenous people was 150% higher than the Brazilian average, while for lethality this value was 6.8% ^29^.

Our analyses demonstrate that illiterate patients had significantly higher in-hospital mortality rates when compared to patients with higher education. Race/skin color and schooling level are important social determinants and impact access to health care, in addition to being strong predictors of mortality ^15^ ^,^ ^30^.

These findings are consistent with the Brazilian Institute of Geography and Statistics (IBGE) data that demonstrate higher levels of economic and social vulnerability in black, brown and indigenous populations ^31^. The NOIS/PUC-Rio, by relating race/skin color and schooling level, showed that black and brown people, when compared to white people of the same schooling level, presented 37% more deaths, with the largest difference in higher education, with 50% ^32^.

In-hospital mortality rate was also higher in black patients regardless of the nature of the health care institution in which they were hospitalized, but in private institutions the chance of death was even higher. In the pandemic context, the availability of and access to hospital services, the number of public and private beds, ICU beds and mechanical ventilators were decisive for the management of more complex cases and for a favorable outcome. However, these resources are more available to the economically higher social strata, which consist mostly of white individuals in Brazil ^33^.

It should be noted that COVID-19 was responsible for collapsing health care systems, such as the Brazilian Unified National health System (SUS), which had serious problems to manage to serve the entire population, especially in the great peaks of the disease ^34^. It is also noted that considering all the services provided by the system, the black population represents 67% of the public served, compared to 47.2% of the white population, and most of the services are concentrated in users with an income range between one quarter and one half of a salary, showing the dependence of this population in relation to the system ^35^ ^,^ ^36^.

A study that analyzed data from SIVEP-Gripe, in May 2020, reported that the white population was more likely to be admitted to the ICU when hospitalized and had death rates similar to the brown population ^37^. Contrary to this finding, in our analyses, black and brown patients, despite being more admitted to the ICU, had 13% and 24% higher chance of death, respectively, when compared to white people. Among the indigenous population, although they were less admitted to the ICU, they were 47% more likely to die, compared to the other groups.

In the data analysis, we also observed that, in relation to white race/skin color, black, brown and indigenous patients were less submitted to tomography examination - OR = 0.72, 0.67, and 0.85 respectively. These patients also had a higher chance of death, reaching 87% in the indigenous population. Of the patients who underwent chest X-ray, the chance of death was also higher among black, brown and indigenous people, with 22%, 25% and 60% respectively.

These findings allow us to infer not only that these patients were hospitalized late in the ICU and with more worsened health status, but also that the difficulties and obstacles to access health services are still present in the lives of these populations. The maintenance of racism and its various faces is directly reflected in the socioeconomic characteristics and conditions in which black and indigenous people live in Brazil, and such characteristics are directly related to access to health care, in its broader concept ^36^ ^,^ ^38^.

As for the indigenous population, which presented alarming data, COVID-19 exposed not only the inequities previously installed in their living and health conditions, but also the weaknesses of a subsystem designed to provide them with differentiated care in the scope of SUS ^39^ ^,^ ^40^ ^,^ ^41^.

Brazil is a country of major contrasts, and the situation is more challenging among indigenous people living in more remote regions, as the health care infrastructure is precarious and access to municipalities with highly complex care requires travelling at least four hours ^42^. In these regions, the availability of vacant beds is even more limited, making access to intensive care extremely difficult ^43^.

Access to information is also an important factor in this analysis because, according to IBGE, black or brown populations have disadvantage in the indicators of Internet use and ownership of mobile phones for personal use, compared to the white population ^31^.

Regarding the use of ventilatory support, we observed that black, brown and indigenous patients were less submitted to this intervention - OR = 0.89, 0.80 and 0.85 respectively -, in addition to having higher chances of death - 23%, 32% and 80% respectively. Despite evincing the black population’s difficulty as to access to mechanical ventilators, inequalities begin long before being in a hospital bed, being observed in housing conditions, in the spatial distribution of households, and in access to services ^31^.

Most of these people have informal jobs and in essential sectors, who remained active during the pandemic and could not use remote work; they live in urban clusters, with a high number of people per room, often without access to piped water and/or electricity, and cannot adopt social distancing measures ^38^ ^,^ ^44^ ^,^ ^45^.

The race/skin color variable, in addition to impacting access to health care services in the pandemic, was also related to undergoing diagnostic tests for the disease. An ecological study carried out in the city of Rio de Janeiro pointed out that diagnostic tests were carried out in neighborhoods where there is a higher per capita income and a higher incidence of white residents. On the other hand, neighborhoods with a larger black population have fewer tests and positive cases ^33^.

The unequal impact of COVID-19 on both the black and indigenous populations is not surprising, as the pandemic intensified preexisting vulnerabilities, further exposing these populations to the new coronavirus ^44^ ^,^ ^46^. It is understood that a causal relation between race/skin color and the emergence of diseases is not established, but it is noted that this information can provide significant indications about the living and health conditions of these groups ^47^.

In terms of the analysis period, we observed a decrease in racial disparities in COVID-19 deaths from 2020 to 2021, but which increased in 2022. We can infer that the decrease in deaths in this period can be justified by the implementation of vaccination against COVID-19 in Brazil in January 2021 ^48^. National and international studies observed high effectiveness of the vaccines in reducing severe cases of the disease, the number of hospitalizations and, consequently, mortality ^49^.

On the other hand, 2022 saw the rapid proliferation of the Omicron variant, which has high transmissibility and caused a resurgence of the pandemic, interrupting a downward trend in the number of cases and deaths caused by SARS-CoV-2 ^50^. It is possible to infer that the rapid transmission of Omicron, combined with the maintenance of inequities in access to health care/vaccination among the black population, justify the increase in racial disparities in terms of deaths from COVID-19 in 2022. It should be noted that international studies indicate that the risk of reinfection with COVID-19 by Omicron is six times higher among unvaccinated people and that most new hospitalizations due to the variant are also concentrated in this group ^50^ ^,^ ^51^.

In Brazil, vulnerable populations are the most impacted by COVID-19 and require special attention ^52^ ^,^ ^53^ ^,^ ^54^. We note a series of omissions and disorganizations by the government, in addition to a dubious conduct of the Federal Government in the fight against the pandemic, as only in April 2020 the Brazilian Ministry of Health included information on race/skin color in the the epidemiological reports of COVID-19, after pressure from black movements, professional associations and scientific associations ^44^ ^,^ ^55^.

The lack or inadequate completion of such information can be interpreted as the subjectivity of racism and the resistance to changes in insufficient practices to guarantee health for these groups ^38^. Health records are strategic and fundamental for learning about the morbidity and mortality conditions of populations and for the decision-making of government managers ^41^.

Government support for the income of low-income families, access to diagnostic tests, emphasis on home care, provision of shelter for the homeless and improved access to health care, by strengthening the SUS and all its instances, have the potential to improve this current situation ^52^ ^,^ ^53^ ^,^ ^54^.

As limitations of this study, we cannot guarantee that all cases hospitalized in Brazil have been included, although notifications of hospitalizations due to COVID-19 in the SIVEP-Gripe system are mandatory. In addition, a significant amount of data was presented as “not informed” due to data collection and manual input into the system. However, the national surveillance system is the main repository of COVID-19 hospitalizations throughout the country and a large amount of information was surveyed for a long period during the pandemic.

## Conclusion

Racial differences were observed in the use of health care services and in outcomes of in-hospital death from COVID-19. Among Brazilian adults hospitalized with SARS/COVID-19, black, brown and indigenous patients had higher in-hospital mortality and lower use of hospital resources. black, brown and indigenous race/skin color populations have severe disadvantages compared to white people and racism and social inequities, which are historical in Brazil, have been aggravated in the context of the COVID-19 pandemic.

The insistence in denying basic and fundamental rights has characterized a racist structure that has operated the policy to combat COVID-19 in the country, as well as extending to other public health problems. Overcoming this structure requires expanding the government’s dialogue with society and health care professionals, in addition to building and enforcing public policies to combat racism to mitigate this historical legacy, which existed before the COVID-19 pandemic and which was further aggravated during it.

## References

[1] Souza ASR, Amorim MMR (2021). Mortalidade materna pela COVID-19 no Brasil. Rev Bras Saúde Mater Infant.

[2] Secretaria de Vigilância em Saúde.Ministério da Saúde (2022). Guia de vigilância epidemiológica: emergência de saúde pública de
importância nacional pela doença pelo coronavírus 2019.

[3] World Health Organization WHO coronavirus (COVID-19) dashboard..

[4] Organização Pan-Americana da Saúde Excesso de mortalidade associado à pandemia de COVID-19 foi de 14,9
milhões em 2020 e 2021..

[5] (2021). Tracking COVID-19 excess deaths across countries.. The Economist.

[6] Aldridge RW, Lewer D, Katikireddi SV, Mathur R, Pathak N, Burns R (2020). Black, Asian and minority ethnic groups in England are at
increased risk of death from COVID-19 indirect standardisation of NHS
mortality data. Wellcome Open Res.

[7] Mackey K, Ayers CK, Kondo KK, Saha S, Advani SM, Young S (2021). Racial and ethnic disparities in COVID-19-related infections,
hospitalizations, and deaths a systematic review. Ann Intern Med.

[8] Golestaneh L, Neugarten J, Fisher M, Billett HH, Gil MR, Johns T (2020). The association of race and COVID-19 mortality. EClinicalMedicine.

[9] Batty GD, Gaye B, Gale C, Hamer M, Lassale C (2021). Explaining ethnic disparities in COVID-19 mortality:
population-based, prospective cohort study.. medRxiv.

[10] Ministério da Saúde Painel Coronavírus..

[11] Secretaria de Políticas de Saúde.Ministério da Saúde (2001). Manual de doenças mais importantes, por razões étnicas, na população
brasileira afro-descendente.

[12] Araújo ME, Caldwell KL, Pereira M, Santos A, Magalhães IS, Ferreira PL (2021). Morbimortalidade pela COVID-19 segundo raça/cor/etnia a
experiência do Brasil e dos Estados Unidos. Saúde Debate.

[13] Giovanatti A, Elassar H, Karabon P, Wunderlich-Barillas T, Halalau A (2021). Social determinants of health correlating with mechanical
ventilation of COVID-19 patients a multi-center observational
study. Int J Gen Med.

[14] Yancy CW (2020). COVID-19 and African Americans. JAMA.

[15] Peres IT, Bastos LSL, Gelli JGM, Marchesi JF, Dantas LF, Antunes BBP (2021). Sociodemographic factors associated with COVID-19 in-hospital
mortality in Brazil. Public Health.

[16] Portal Brasileiro de Dados Abertos SRAG 2020. Banco de dados de síndrome respiratória aguda grave -
incluindo dados da COVID-19..

[17] Bahia L, Scheffer M (2018). O SUS e o setor privado assistencial interpretações e
fatos. Saúde Debate.

[18] Almeida S (2018). O que é o racismo estrutural?.

[19] Machado K O racismo em três séculos de escravidão..

[20] Silva NN, Favacho VBC, Boska GA, Andrade EC, Merce NP, Oliveira MAF (2020). Acesso da população negra a serviços de saúde revisão
integrativa. Rev Bras Enferm.

[21] Santos RV, Escobar AL (2001). Saúde dos povos indígenas no Brasil perspectivas
atuais. Cad Saúde Pública.

[22] Instituto Brasileiro de Geografia e Estatística (2012). Os indígenas no Censo Demográfico 2010: primeiras considerações com base
no quesito raça/cor ou raça.

[23] Lana RM, Codeco CT, Santos RV, Cunha B, Coelho FV, Cruz OG, Freitas CM, Barcellos C, Villela DAM (2021). COVID-19 no Brasil: cenários epidemiológicos e vigilância em
saúde.

[24] Ministério da Saúde (2002). Política Nacional de Atenção à Saúde dos Povos Indígenas..

[25] Brito CAG A história da saúde indígena no Brasil e os desafios da pandemia de
COVID-19..

[26] Maggi RS (2014). A saúde indígena no Brasil. Rev Bras Saúde Mater Infant.

[27] Evangelista AP Negros são os que mais morrem por COVID-19 e os que menos recebe vacinas
no Brasil escravidão..

[28] Diferenças sociais: pretos e pardos morrem mais de COVID-19 do que
brancos, segundo NT11 do NOIS..

[29] Assessoria de Comunicação, Conselho Nacional de Saúde Divergência de dados sobre COVID-19 na população indígena dificulta
medidas efetivas de proteção..

[30] Abedi V, Olulana O, Avula V, Chaudhary D, Khan A, Shahjouei S (2021). Racial, economic, and health inequality and COVID-19 infection in
the United States. J Racial Ethn Health Disparities.

[31] Instituto Brasileiro de Geografia e Estatística Desigualdades sociais por cor ou raça no Brasil..

[32] Lima IC, Sabino GFT Anais do VI Congresso Nacional em Educação..

[33] Dantas MNP, Silva MFS, Barbosa IR (2022). View of reflections on the COVID-19 mortality among the black
population and racial inequality in Brazil. Saúde Soc.

[34] MonitoraCovid-19 (2021). O "represamento" do atendimento em saúde no SUS. Relatório
final..

[35] Instituto de Pesquisa Econômica Aplicada (2021). Atlas da violência 2021.

[36] Secretaria de Gestão Estratégica e Participativa, Ministério da
Saúde (2017). Política Nacional de Saúde Integral da População Negra: uma política
para o SUS..

[37] Baqui P, Bica I, Marra V, Ercole A, Van der Schaar M (2020). Ethnic and regional variations in hospital mortality from
COVID-19 in Brazil a cross-sectional observational study. Lancet Glob Health.

[38] Batista LE, Proença A, Silva A (2021). COVID-19 e a população negra. Interface (Botucatu).

[39] Associação Brasileira de Saúde Coletiva (2020). A COVID-19 e os povos indígenas: desafios e medidas para controle do seu
avanço.

[40] Mota SEC, Scalco N, Pedrana L, Almeida A, Barreto ML, Pinto EP, Aragão E, Barral-Netto M (2020). Construção de conhecimento no curso da pandemia de COVID-19: aspectos
biomédicos, clínico-assistenciais, epidemiológicos e sociais..

[41] Oliveira RG, Cunha AP, Gadelha AGS, Carpio CG, Oliveira RB, Corrêa RM (2020). Racial inequalities and death on the horizon COVID-19 and
structural racism. Cad Saúde Pública.

[42] Fundação Oswaldo Cruz (2020). Regiões e Redes Covid-19: acesso aos serviços de saúde e fluxo de
deslocamento de pacientes em busca de internação. Relatório final.

[43] Matta GC, Rego S, Souto EP, Segata J (2021). Os impactos sociais da COVID-19 no Brasil: populações vulnerabilizadas e
respostas à pandemia.

[44] Associação Brasileira de Saúde Coletiva (2021). População negra e COVID-19.

[45] Oliveira LM, Wilvert JM (2022). Fatores associados à morte por COVID-19 na população negra no
Brasil.

[46] Silva SA (2021). A pandemia de COVID-19 no Brasil: a pobreza e a vulnerabilidade
social como determinantes sociais.. Confins.

[47] Braz RM, Oliveira PTR, Reis AT, Machado NMS (2013). Avaliação da completude da variável raça/cor nos sistemas
nacionais de informação em saúde para aferição da equidade étnico-racial em
indicadores usados pelo Índice de Desempenho do Sistema Único de
Saúde. Saúde Debate.

[48] Leonel F Brasil celebra um ano da vacina contra a COVID-19..

[49] Gameiro N Estudo aponta aumento da eficácia da vacina de COVID-19 em mais de 90%
com dose de reforço..

[50] Seis fatos sobre a ômicron, a variante mais transmissível da
COVID-19..

[51] Conselho Nacional de Saúde Frente Pela Vida defende vacinação infantil: "O Brasil precisa proteger
suas crianças"..

[52] Abrams EM, Szefler SJ (2020). COVID-19 and the impact of social determinants of
health. Lancet Respir Med.

[53] Lee A, Morling J (2020). COVID19 the need for public health in a time of
emergency. Public Health.

[54] ONU Mulheres Brasil "Ações de enfrentamento à pandemia devem considerar condição de vida e
saúde de negras e negros", diz sanitarista à ONU Mulheres Brasil..

[55] Santos HLPC, Maciel FBM, Santos KR, Conceição CDVS, Oliveira RS, Silva NRF (2020). Necropolitics and the impact of COVID-19 on the black community
in Brazil a literature review and a document analysis. Ciênc Saúde Colet.

